# Indents

**Published:** 1910-03-15

**Authors:** 


### INDENTS.

☞Brief Articles of Technical or Scientific Value will receive notice and comment. Contributions invited.

*A Method of Repairing Rubber Plates.* This is a simple method of replacing teeth on plates by making grooves and undercuts, and after applying soldering fluid to pins, flow Melotte's metal around the pins and fill up the cavity and undercuts flush with the plate. Then polish down even with the plate. The metal is manipulated with a hot spatula.

This method you will find quite a time saver and a more satisfactory means of repair than by the old way.—Dr. J. P. Gray in *Summary.*

*To Prevent Dark Spaces in Plates.* To overcome dark spaces in plates I use *white* rubber between the teeth, placing it carefully just where I want it when the case is complete. I then pack pink rubber over this and around the teeth as usual, and vulcanize and finish in the customary way.

When the plate is polished the white rubber prevents the shadows that so often produce those unsightly dark spaces. The work must be done carefully to get the best results.—Dr. A. C. Barclay in *Summary.*

*Mastication With Full Dentures.* In the use of artificial teeth, full sets, the patient does not grind, but uses a down pressure. This is especially true in the case of flat, narrow, lower jaws, where it would be utterly impossible to grind without shoving the lower denture sideways. I speak from personal experience of twelve years with this condition. I may say incidentally that by the experience I have had with my own I have learned things in regard to lower dentures that I could not have learned from others.—L. P. Haskell, D.D.S., Chicago, *The Dental Digest*, December, 1909.

*Gold Fillings in Porcelain Teeth.* To put a gold filling in a mineral tooth, grind a depression in the tooth where the filling is required, then cover the ground surface with a little Jenkins inlay, or other low-fusing body, and on this press a piece of sponge gold the same size. Fuse this in furnace, and then condense gently with engine mallet, add gold and finish in the same way as with a gold filling. If required, a tooth may be entirely faced with gold in this manner, giving the appearance of an all-gold crown, the inlay body holding the first layer of gold quite firmly, if ordinary care is taken as with a gold filling.—C. E. Brown in *British Dental Journal.*

*Suggestion about Arranging Artificial Teeth.* You cannot put too much time and trouble on a set of artificial teeth, whether you are properly paid for them or not. If you make them at all they should be made as well and as natural as it is possible to make them. Make them with the idea of restoring what has been lost in that individual through the loss of the teeth. If the patient has lost the teeth from the effects of pyorrhea, irregularity, deformity, or any other cause, the new teeth should not be put in the mouth in a stiff, hard row, but should be made in such a way that they will have a slight suggestion of the way the natural teeth originally were in the mouth, even to the extent of suggesting slightly the trouble which caused the loss of the teeth.—S. C. G. Watkins in *Items of Interest.*

*Zinc Dies.* Dr. A. De Witt Gritman gave an interesting discourse upon prosthetic manipulations, illustrating his remarks with a large number of models, etc. His method of casting zinc dies was new, and produced excellent results. Instead of waiting until all the zinc in the melting pot was fluid, he begins to pour as soon as enough is melted to form the face of the die, then returns the pot to the fire until a little more is melted; this is then poured, and this continued until the die is thick enough. The interval between the several pourings is very short, the additions being made before the zinc previously poured has set. By this means he gets a more solid die, the hole usually seen in the base of zinc dies, caused by shrinkage, is avoided, and the zinc is kept in very much better condition on account of not being overheated. He stated that the dies shown were made of zinc that had been in constant use several years. They were smooth, clean and free from granulation and oxid as though the zinc had been used but once.—*Dental Scrap Book.*

*Dental Plate Work; Its Place Professionally.* There is no department of dentistry that requires a more thorough knowledge of anatomy, a better understanding of physiological processes and pathological conditions, or a higher artistic training than dental plate work, if that work is done as well as it is possible to do it. Artistic plate work has robbed old age of its loose-lipped hideousness; it has added years to life by restoring, in part at least, the function of mastication; it has added largely to the sum total of human happiness by imparting the consciousness of improved appearance. And to do these things and to do them well is just as professional, ethical and dignified as is the administration of drugs, the hammering of gold, or the wielding of the surgeon's knife.—F. G. Worthley, D.D.S., *Western Dental Journal,* December, 1909, page 927.

*Details to be Observed in Swaging Metal Plates.* Be sure to take the impression with the compound of plaster, pumice and whiting as described in my former article; take fine talcum powder and spread over the impression, then a wad of cotton and wipe the talcum into the fine pores of the impression, which will give a smooth surface. Place the impression directly over the center of the base block in such a manner that the model or die will be not less than one-half inch thick in the palate. Be very careful to build up around the edge of the impression on the tray with the pumice and plaster compound so that the metal will not run through and so that the model will fill out full at the heels. Heat the base block and impression to the same temperature as the fusing point of the die metal. Do not heat the die metal too hot, but just hot enough to pour, and pour carefully and quickly. Be sure the metal has cooled before separating from impression, to avoid fracture. After separating, carefully oil the die with glycerine and select a blank plate to conform fairly well to the model or die. See that it is annealed, then cover the die with a piece of soft muslin, take another piece of muslin and fold several times to spread over the plate, then take the horn mallet and tap over the folded muslin to conform the plate to the die; anneal carefully and place in the press.

Be sure to place a sheet of rubber dam over the plate slightly larger than the plate to prevent the rubber counter die material from getting between the plate and die; place in the press and turn down only moderately hard, remove and if the plate is buckling tap it out with horn mallet, anneal and place in press. Now apply heavy pressure; remove and trim plate to size desired; try in the mouth and if the fit is right place on die and take line graver one-sixteenth inch wide and cut spurs for retaining rubber, anneal and place in press to see that the plate fits perfectly, then apply wax and get the bite; but before trying plate with wax in the mouth the palate of the plate must be cleaned with a saturate solution of caustic soda and rinsed in pure water. A groove should be cut across the die at back part of plate so the edge of the plate will press slightly into the soft tissues, but must not rest heavy on the hard plate. By using the muslin in conforming the plate to the die, the plate will not be bruised as by the old methods, but will retain the original polish.—W. T. Wallace, in *Items of Interest.*

*A Peculiar Conditon—How to Meet it.*

What are the prime requisites of an upper artificial denture? A perfect fitting plate, fair adhesion, proper occlusion and proper arrangement of the teeth and artificial gums to restore the features to normal condition.

What is a proper occlusion? Exact pressure on the bicuspids and first molars, the second molars not quite in contact, and no contact in any case of the anterior teeth.

Why no contact of the anterior teeth? Two reasons: First, contact with the upper teeth in the usual overbite tends to displace the plate, as the leverage is so great from the tips of the teeth to the rear of the plate. Second, undue pressure across the anterior margin of jaw causes undue absorption of the bone, leaving what so often is seen, a flexible ridge.

A peculiar condition at times occurs, difficult to overcome, and for which there is but one successful solution. Remaining on the lower jaw are the anterior teeth and two or three roots upon which are crowns. Some dentists advise the retention of the roots and crowns. What results? The pressure is on the anterior and left posterior teeth, covering two-thirds of the upper jaw with no pressure upon the right third. The denture is not balanced and sooner or later the bone is lowering, especially in front; the plate becomes a misfit and loosens.

What is the remedy? Some say a partial lower. That is all right so long as it remains intact, but it is only a question of a short time when it settles, and the pressure reverts to the front and left side. The proper thing to do, in order to secure permanent and satisfactory results, would be to extract roots on the left side, put in a temporary partial lower, setting the teeth high enough to well clear the anterior teeth; then at the end of the year, or sooner, replace with a permanent denture; thus the upper denture is evenly balanced and absorptions arrested.

With regard to the matter of extracting certain teeth, no matter how sound, the only question should be "What shall be done to make the denture the most comfortable and useful?"—Dr. L. P. Haskell in *Items of Interest.*

*Dr. Jonnesco and "Stovain."* The writers of the daily press have had much to say recently of a new method of anesthesia introduced into this country by a Dr. Jonnesco, a Roumanian physician. The new method, we are confidantly told, will make it possible for the patient to sit up, smoke a cigar, and converse with the surgeon who is cutting off his leg, since the brain is not affected by the anesthetic. The method is thus what is usually called "local anesthesia," since it does not affect the whole system; but it is applied to much larger regions than has usually been the case with such methods, which have been used to render insensible a finger, an eye, or some other small part of the body. In this widened method of localization the anesthetic is not applied directly, but is injected into the spinal cord—a plan by no means new, as it was described in these pages several years ago. It has, however, been regarded as dangerous by most surgeons. The danger has now been obviated, we are assured by Dr. Jonnesco, by the admixture of strychnin in proper proportions with the anesthetic. The drug used by Jonnesco is stovain—one of the so-called "cocain substitutes." Says an editorial writer in *The Medical Record* (New York, November 27):

"The use of arachnoid injections of one of the cocain substitutes as a means of inducing general anesthesia has not found great favor among surgeons in this country, although a few have employed them and some have advocated them strongly. The fact that this method could be used only when the lower part of the body was the seat of operation, injection into the upper part of the cord being rightly looked upon as dangerous, has had much to do with the disfavor with which the method was regarded. The feeling has been that, the danger of upper spinal injections being admitted, there was no guaranty that the influence of low injections might not extend upward to the bulbar region. Hence there was little disposition to flee from the familiar dangers of inhalation-anesthesia to the unknown perils of spinal injection.

"General spinal anesthesia has, however, had its enthusiastic advocates in France and elsewhere on the Continent, and at the International Congress of Surgery in Brussels in September, 1908, Jonnesco, of Bucharest, read a paper advocating this method for operations in the upper as well as in the lower part of the body, claiming that the addition of strychnin to the anesthetic deprived the procedure of all danger even when injection was made into the arachnoid of the upper dorsal spine. The paper was not particularly well received, and Bier, of Berlin, Rehn, of Frankfort, and others in Germany have warned their colleagues against a resort to what they consider a dangerous measure. In answer to their condemnation, Jonnesco went to London and is coming to this country to show by practical demonstration in the operating-room that his method is not only safe but possesses numerous advantages over inhalation-anaesthesia.

"In *The British Medical Journal* of November 13, Jonnesco writes that since October, 1908, he has used spinal anesthesia in all his operations, hospital and private, never once having recourse to anesthesia by inhalation, and his hospital colleagues have also employed the method with complete success."

The novelty of Jonnesco's method consists, we are told, in the prevention of untoward symptoms by the use of strychnin, as already noted, owing to which the anesthetic may be applied either to the lower or the upper part of the spine, according to the region in which the operation is to take place. We read:

"Among the advantages which the writer claims for general spinal anesthesia are that it can be given by the surgeon himself, thus doing away with the need of a special anesthetist and so reducing the number of assistants ; that it can be used in any case with absolute safety, there being no contraindication to its employment; that it greatly simplifies operations on the face or throat by doing away with the troublesome mask; that it usually is attended by immobility of the limbs by reason of the paresis resulting from the anesthesia of the spine; and finally that there is immobility of the abdominal viscera, including the intestines, the advantage of which in cases of laparotomy can hardly be overestimated.

"Concerning the safety of the method Jonnesco speaks with the most absolute confidence, with a disquieting confidence indeed, for it is not founded on a sufficiently long or wide experience. He has used it in . . . a total of 623 operations in the course of a year, without a death and without any serious complications . . . That is indeed a strong showing and explains, even if it does not entirely justify, the author's confidence. The cable reports that he has overcome the conservatism of the London surgeons and has demonstrated the method in some of the hospitals in that city, and it adds that he will soon come to America to urge the practice of general spinal anesthesia upon his professional brethern here."

We find the following account of an operation by Professor Jonnesco at the Seamen's Hospital at Greenwich, England, in the London *Daily Mail* for November 19:

"When the surgeons' hands were fully sterilized for the operation, Professor Jonnesco inserted a small hypodermic needle into the spinal canal, passing it between two of the vertebrae at the base of the neck. Attaching a small syringe to the needle, 3 centigrams of stovain and 5 centigrams of sulphate of strychnin dissolved in water weie injected into the spinal canal. After one minute the patient was told to lie down on the operating table, and his head and shoulders were lowered, so that the action of gravity would cause the numbing fluid to spread upward.

"Two minutes later the operation, which was for the removal of a mass of tubercular glands in the neck, was carried out in the ordinary manner, the patient perfectly conscious and talking to the surgeons during the whole proceedings.

"Do you feel any pain?" asked one of the surgeons. "No, sir," said the patient.

"Are you quite comfortable?"

"Yes, thank you," said the man.

"The uncanny effect of these words spoken by a man with a gaping wound three inches long and an inch deep in his neck can hardly be described.

"Five minutes after the bandages were applied the patient got off the operating table and walked into the next room to the stretcher which was to carry him back to his ward."—*Literary Digest.*

